# Structural Characterization of Two CO Molecules Bound to the Nitrogenase Active Site

**DOI:** 10.1002/anie.202015751

**Published:** 2021-01-27

**Authors:** Trixia M. Buscagan, Kathryn A. Perez, Ailiena O. Maggiolo, Douglas C. Rees, Thomas Spatzal

**Affiliations:** ^1^ Division of Chemistry and Chemical Engineering California Institute of Technology 1200 E. California Blvd. Pasadena CA 91125 USA; ^2^ Howard Hughes Medical Institute California Institute of Technology 1200 E. California Blvd. Pasadena CA 91125 USA; ^3^ Present address: European Molecular Biology Laboratory Meyerhofstrasse 1 69117 Heidelberg Germany

**Keywords:** carbonyl ligands, C−C coupling, cofactors, nitrogenases, X-ray diffraction

## Abstract

As an approach towards unraveling the nitrogenase mechanism, we have studied the binding of CO to the active‐site FeMo‐cofactor. CO is not only an inhibitor of nitrogenase, but it is also a substrate, undergoing reduction to hydrocarbons (Fischer–Tropsch‐type chemistry). The C−C bond forming capabilities of nitrogenase suggest that multiple CO or CO‐derived ligands bind to the active site. Herein, we report a crystal structure with two CO ligands coordinated to the FeMo‐cofactor of the molybdenum nitrogenase at 1.33 Å resolution. In addition to the previously observed bridging CO ligand between Fe2 and Fe6 of the FeMo‐cofactor, a new ligand binding mode is revealed through a second CO ligand coordinated terminally to Fe6. While the relevance of this state to nitrogenase‐catalyzed reactions remains to be established, it highlights the privileged roles for Fe2 and Fe6 in ligand binding, with multiple coordination modes available depending on the ligand and reaction conditions.

## Graphical abstract

Besides reducing dinitrogen, nitrogenases reduce CO to hydrocarbons, mediating C−C bond formation between multiple CO‐derived ligands. To shed light on how multiple C‐based ligands bind to the active site, we report a 1.33 Å resolution crystal structure of two CO ligands coordinated to the FeMo‐cofactor of molybdenum nitrogenase (see picture). The second CO ligand is coordinated to Fe6 in a previously unreported ligand binding mode.

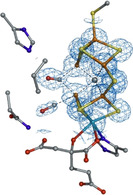

Biological nitrogen (N_2_) fixation is catalyzed by nitrogenases (N_2_ases). The most well‐studied N_2_ase (molybdenum [Mo] N_2_ase) consists of two component proteins: the Fe protein, a homodimer which contains a [4Fe4S] cluster, and the MoFe protein, a heterotetramer which contains two unique complex metalloclusters per heterodimer.[^1^, ^2^, ^3^, ^4^, ^5^] During catalysis, the two component proteins form a complex, promoting ATP‐dependent electron transfer from the Fe protein to the MoFe protein.^[3]^ Substrate reduction ultimately occurs at the multimetallic active site of the MoFe protein, called the FeMo‐cofactor, by a mechanism that remains enigmatic.^[6]^ The FeMo‐cofactor is comprised of iron and molybdenum atoms with an overall composition of [7Fe:9S:1C:1Mo]–*R*‐homocitrate.^[7]^ Alternative N_2_ases, featuring cofactors with V or Fe in place of the Mo ion, are expressed under Mo‐deficient conditions.[^4^, ^8^, ^9^]

The as‐isolated state of the Mo N_2_ase active site does not bind substrates, implying that the active site must be activated for substrate binding.[^6^, ^10^] Indeed, it has long been proposed that the N_2_ase active site features multiple substrate binding sites and that the formation of these binding sites requires the particular substrate under turnover conditions; that is, the cofactor is dynamic during catalysis.[^11^, ^12^] Given the complexity of the N_2_ase active site and the lack of ligand binding to the as‐isolated [7Fe:9S:1C:1Mo]–*R*‐homocitrate cofactor form, the nature of substrate (or inhibitor) coordination remained elusive until defined in detail by high resolution structural studies of ligand‐bound Mo and vanadium [V] N_2_ases.[^2^, ^10^, ^13^, ^14^, ^15^, ^16^] Evidence for the dynamic behavior of the cofactor was provided by the observation that selenium could be substituted into a specialized group of sulfurs in the FeMo‐cofactor known as the belt sulfides, and could migrate through these positions under turnover conditions.^[13]^

Given the ability of N_2_ases to catalyze CO reduction to hydrocarbons (Fischer–Tropsch‐type chemistry),[^17^, ^18^, ^19^] it is important to structurally characterize various CO binding modes at the active site since this information could help illuminate the mechanism of hydrocarbon formation at an atomic level. In particular, for Mo N_2_ase, methane (CH_4_) was not detected as a product of CO reduction; rather, higher order hydrocarbons were detected, suggesting that multiple CO‐derived molecules could bind to the FeMo‐cofactor at a time.^[17]^ We reported the initial crystal structure of a ligand bound form of Mo N_2_ase from *Azotobacter vinelandii* (Av) in which one of the belt sulfides, S2B, of the cofactor is displaced by a carbon monoxide (CO) molecule (**Av1‐CO**);^[10]^ a similar binding mode was subsequently demonstrated for the Av vanadium nitrogenase.^[16]^ Spectroscopic studies have highlighted that several distinct CO‐bound species can be observed under turnover conditions.[^20^, ^21^, ^22^, ^23^] As the CO‐binding site(s), the nature of CO‐binding, and the possibility of S2B displacement could not be unequivocally established from the spectroscopic data,[^24^, ^25^] it became of interest to determine how multiple CO molecules might bind to the FeMo‐cofactor. In this study we extend our previously established procedure to structurally characterize ligand‐bound and sulfide‐substituted states of the FeMo‐cofactor with an approach that resulted in two CO molecules trapped at the active site.

The crystal structure solved at 1.33 Å resolution of a new CO‐bound state of the MoFe protein, **Av1(CO)_2_**, is shown in Figure 1. **Av1(CO)_2_** was challenging to prepare due to the weaker binding of the second CO and required pressurization of **Av1‐CO** crystals to 80 psi to maximize occupancy of that site. It is important to note that **Av1(CO)_2_** can only be observed when **Av1‐CO** is used as the starting material; that is, FeMo‐cofactor in the as‐isolated Av1 protein is incapable of binding CO on its own, consistent with previously reported studies.[^1^, ^10^] In the **Av1(CO)_2_** structure, one CO ligand is bridged between Fe2 and Fe6 in an analogous fashion to the previously reported **Av1‐CO** structure while a second CO ligand is terminally bound to Fe6 (Figure 1 A). The proximity of the two CO ligands is intriguing, especially in the context of C−C coupling at the Mo N_2_ase active site. Indeed, mechanistic proposals for C_2+_ hydrocarbon formation include mono‐ or bimetallic reductive elimination of CO‐derived alkyl ligands at one metal center or between neighboring metal centers. Alternatively, migratory insertion mechanisms between CO and CO‐derived alkyl ligands are feasible.^[26]^ Our structural data suggest that these mechanistic proposals are possible as Fe6 can accommodate two CO ligands and may serve as either a single site for C−C bond formation or in a dinuclear site in cooperation with Fe2.

**Figure 1:**
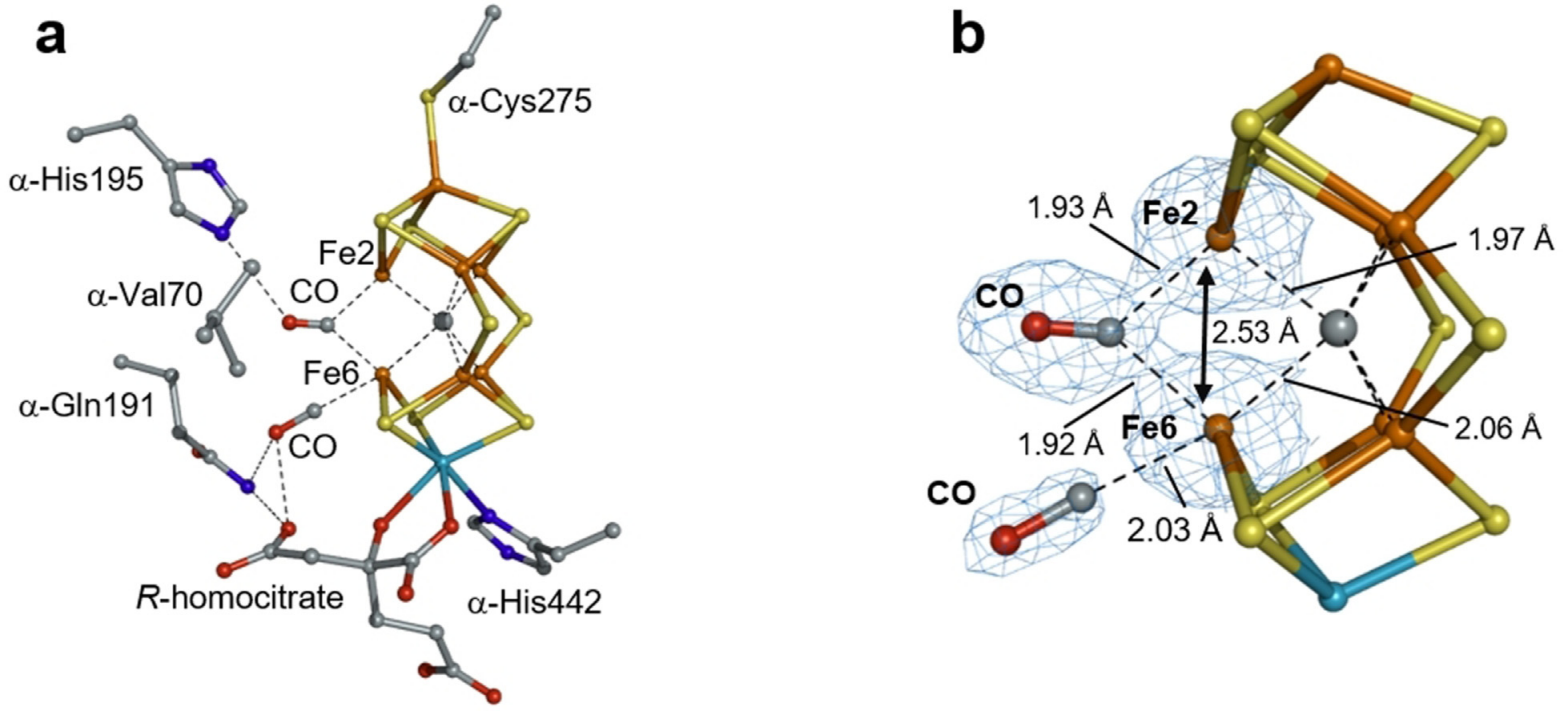
The FeMo‐cofactor with two bound CO ligands [**Av1(CO)_2_**]. Refined structure of **Av1(CO)_2_** in the vicinity of the FeMo‐cofactor at a resolution of 1.33 Å. a) Side‐view of the FeMo‐cofactor highlighting the two CO ligands and the protein environment near the CO ligands. b) Magnified view of the FeMo‐cofactor in chain A with overlaid electron density (2 *F*_obs_−*F*_calc_) map surrounding Fe2, Fe6, and CO atoms contoured at 1.0 σ (represented as a blue mesh). Selected bond distances are shown. Iron atoms are shown in orange, sulfur in yellow, molybdenum in turquoise, carbon in gray, nitrogen in blue, and oxygen in red.

In this structure, Fe6 adopts a five‐coordinate trigonal bipyramidal geometry as opposed to the four‐coordinate tetrahedral geometry observed for the other Fe centers in this structure (including Fe2), as well as in the structures of the as‐isolated or **Av1‐CO** crystal structures.[^7^, ^10^] The terminal CO oxygen atom interacts with the side chain amide N of α‐Gln191 (ca. 3.2 Å), which may help stabilize substrate binding at Fe6. Mutagenesis studies of α‐Gln191 suggests the identity of this residue affects ligand coordination and product speciation in CO reduction reactions.^[18]^ Additionally, the terminal CO ligand is near the homocitrate moiety, and previous work has shown that substitutions of the homocitrate with structurally similar analogues decrease the substrate reduction activity.[^27^, ^28^]

While the bridging CO ligand exhibits 100 % occupancy at both cofactors in the heterotetramer, the terminal CO moiety exhibits approximately 50 % occupancy, consistent with an expected weaker association of the second CO molecule under the experimental conditions. The presence of the terminal CO was further validated through inspection of polder omit maps calculated for this ligand (see SI, Figure S1).^[29]^ In **Av1(CO)_2_**, the terminal CO ligands exhibit higher B‐factors relative to the bridging COs, (see SI, Table S2 and Figure S2). One explanation for this observation involves a more dynamic association of the terminal CO, which would result in greater positional displacements and higher B‐factors. A second explanation for the higher B‐factors is that the occupancy could be less than what is currently modeled. Indeed, the reduced occupancy of the terminal CO likely reflects partial dissociation caused by the depressurization that necessarily occurs during cryo‐protection of the crystal in preparation for X‐ray diffraction data collection. Because occupancies and B‐factors are correlated, we cannot distinguish between these two models.^[30]^ With the exception of Fe6, the distances between the interstitial carbon and the Fe in the surrounding trigonal prism average (1.99±0.02) Å (Figure 1 B), close to that observed for the as‐isolated 3U7Q and **Av1‐CO** structures ((2.00±0.01) Å and (1.99±0.02) Å, respectively). The Fe6‐interstital carbon distance increases by approximately 0.06 Å on the binding of the second CO ligand, which is likely an underestimate due to the fractional occupancy of the terminal CO ligand. Although the increase of approximately 0.06 Å is comparable to the estimated coordinate uncertainties (0.040 Å and 0.055 Å, for **Av1(CO)_2_** and **Av1‐CO**, respectively),[^31^, ^32^] it is consistent with observations from synthetic studies in which changes to the Fe geometry are enabled by a flexible Fe–X interaction, thus allowing the Fe center to stabilize π‐basic and π‐acidic species trans to ligand X.[^33^, ^34^, ^35^, ^36^]

To complement our structural data, we obtained EPR spectroscopic data of the **Av1(CO)_2_** pre‐ and postcrystallization (solution and crystal slurry, respectively; Figure 2 and SI). We observed that the solution behavior was consistent with previously reported EPR studies with both hi‐ and lo‐CO species detected (with *g*=2.17, 2.06 and *g*=2.10, 1.98, and 1.92, respectively)[^20^, ^24^, ^37^, ^38^] depending on whether an excess of CO was present upon freezing the protein for storage and during EPR sample preparation. Finally, and in line with our crystallographic data, the EPR spectrum of a crystal slurry consisting of **Av1(CO)_2_** crystals confirms the presence of hi‐CO. The observation of the hi‐CO EPR signal derived from the addition of CO to crystallized **Av1‐CO** is consistent with previous reports in which lo‐CO can be converted to hi‐CO in the absence of turnover conditions.^[21]^

**Figure 2:**
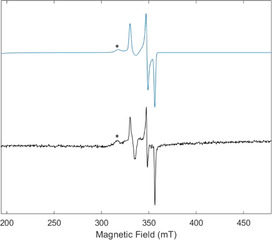
EPR spectrum of a crystal slurry containing **Av1(CO)_2_**. Experimental data (black) and simulation (blue). For the full spectrum and simulation parameters, see the Supporting Information. *Indicates the signal diagnostic for previously reported hi‐CO species.

The Fischer–Tropsch‐type chemistry exhibited by nitrogenase suggests that the multimetallic active site can bind more than one ligand simultaneously. The expansion of the Fe6 coordination environment to accommodate a second CO ligand represents a new mode of FeMo‐cofactor ligand binding that complements the displacement of belt sulfurs by ligands that has been previously reported for Mo and V N_2_ases.[^10^, ^13^, ^15^, ^16^, ^39^] For CO reduction to hydrocarbons, one might intuit that binding of coupled substrates would occur at one metal center or adjacent metal centers, leading to reductive elimination of the hydrocarbon product. At least for the first CO binding event at the cofactor, it has been proposed that CO binds to the more oxidized face of the as‐isolated state of the cluster (as determined by spatially resolved anomalous dispersion on the as‐isolated state of the MoFe protein),^[40]^ which presumably becomes reduced under turnover conditions. On the other hand, binding of the second CO ligand does not require a change to the total oxidation state of the FeMo‐cofactor (although there could be internal redox changes).^[41]^ The binding of a terminal CO ligand to Fe6 is consistent with mutagenesis and spectroscopic studies implicating Fe6 as a site for substrate binding.[^18^, ^25^] We speculate that hydrogen bonding interactions with Gln191 may promote CO binding at Fe6.

Previously reported ^13/12^CO labeling studies by the Ribbe and Hu groups suggest that the hi‐CO form of V nitrogenase is not a competent intermediate in CO coupling, while the lo‐CO form is catalytically competent.^[42]^ Based on these results and others,[^43^, ^44^] a mechanistic hypothesis regarding CO reduction has been proposed in which the first CO ligand is reduced (at least partially) before a second CO molecule can bind to the cofactor and undergo productive reduction.[^4^, ^45^, ^46^] While V and Mo N_2_ase are structurally similar, they do exhibit disparate activities towards CO reduction with the former being much more reactive towards hydrocarbon formation. Given these differences, it is entirely possible that the two nitrogenases follow distinct mechanistic paths for CO reduction.

The FeMo‐cofactor with two bound CO ligands may provide a snapshot of how nature arranges CO‐derived ligands for Fischer–Tropsch‐type chemistry. In particular, Fe2 and Fe6 seem to be preferential sites for binding, with various coordination modes available depending on the identity of the incoming ligand. It is also plausible that similar considerations are relevant for the mechanism of dinitrogen reduction by nitrogenase. Dinitrogen is known to coordinate to mono‐, bi‐ and multimetallic sites via various coordination modes, but only a handful of these dinitrogen complexes lead to productive N_2_ reduction products.[^6^, ^47^] Determining whether certain substrate binding modes are more prone to productive reduction pathways is of particular interest in synthetic inorganic chemistry and now the active site of N_2_ase faces similar questions.

## Conflict of interest

The authors declare no conflict of interest.

## Supporting information

As a service to our authors and readers, this journal provides supporting information supplied by the authors. Such materials are peer reviewed and may be re‐organized for online delivery, but are not copy‐edited or typeset. Technical support issues arising from supporting information (other than missing files) should be addressed to the authors.

Supplementary
