# Supplementary material for: Structural Characterization of Two CO Molecules Bound to the Nitrogenase Active Site
Source: Angew Chem Int Ed Engl. 2021 Jan 27;60(11):5704–7. doi: 10.1002/anie.202015751 (PMC7920927; doi:10.1002/anie.202015751)
Supplement: Supplementary file 1 — Supplementary [file ANIE-60-5704-s001.pdf]

Angewandte Chemie
Eine Zeitschrift der Gesellschaft Deutscher Chemiker

Supporting Information

Structural Characterization of Two CO Molecules Bound to the Nitrogenase Active Site

Trixia M. Buscagan[+], Kathryn A. Perez[+], Ailiena O. Maggiolo, Douglas C. Rees, and Thomas Spatzal**

anie_202015751_sm_miscellaneous_information.pdf

Supporting Information

Table of Contents

Page	Description
S2	Methods
S6	Supplementary **Table S1.** Data collection and refinement statistics for **Av1(CO)$_2$**.
S7	Supplementary **Table S2.** B-factors and occupancies of the CO molecules for **Av1(CO)$_2$**.
S8	Supplementary **Figure S1.** Polder maps of terminal CO ligands.
S9	Supplementary **Figure S2.** Thermal ellipsoid representation at 50% probability of individually refined anisotropic B-factors for cofactor and CO atoms of **Av1(CO)$_2$**.
S10	Supplementary **Figure S3.** Solution spectrum 1 with simulations.
S11	Supplementary **Figure S4.** Solution spectrum 2 with simulations.
S12	Supplementary **Figure S5.** Full crystal slurry spectrum with simulations.
S13	Supplementary **Table S3.** Parameters of EPR simulations.

Methods

General considerations. All protein manipulations were carried out using standard Schlenk or anaerobic tent techniques under an atmosphere of Ar or 97/3% Ar/H_2 mixture, respectively. CO was purchased from Sigma Aldrich. All other reagents were purchased from commercial vendors and used without further purification unless otherwise stated.

Growth of *A. vinelandii* and nitrogenase purification. *Azotobacter vinelandii* growth and nitrogenase purification were performed based on previously published methods with the following modifications.[1,2] All protein buffers were deoxygenated, kept under an argon atmosphere, and contained 5 mM $Na_2S_2O_4$. *In vitro* nitrogenase activity was determined by monitoring acetylene reduction to ethylene as previously described.[3] Ethylene and acetylene quantification was determined using gas chromatography (activated alumina 60/90 mesh column, flame ionization detector). Av1 had a specific activity of 2350 ± 100 nmol min^{-1} mg^{-1} and Av2 had a specific activity of 2050 ± 55 nmol min^{-1} mg^{-1} when measured by acetylene reduction at saturation of each component.

Preparation of Av1-CO. Av1-CO was prepared using a previously reported protocol,[2] with the following modifications. To generate sufficient material for EPR spectroscopy or crystallization, two parallel 12 mL reactions (each containing 1.5 mg of Av1 and 1.65 mg Av2 [component ratio of 2]) were combined and concentrated under an argon overpressure using an Amicon filtration cell with a molecular weight cut-off of 100,000 Da, with intermittent flushing of CO every 2-3 minutes during the concentration. The concentrated mixture was subsequently used for either solution EPR studies or for crystallization experiments. This process was repeated at least three times for all of the data in the paper.

Preparation, crystallization and data collection of Av1(CO)$_2$. Av1-CO was crystallized by the sitting-drop vapor diffusion method at ambient temperature under the strict exclusion of dioxygen in an inert gas chamber containing a 97/3% mixture of N_2/H_2 with < 1 ppm of O_2 using a previously reported protocol.[2] The looped crystals were carefully transferred to a homemade gas pressurization device[4] and pressurized at 80 psi of CO for 2 min resulting in **Av1(CO)$_2$**, after which they were quickly transferred to liquid nitrogen for storing until shipment. Diffraction data were collected at 12,400 eV (0.99987 Å) and 7100 eV (1.74626 Å) at the Synchrotron Radiation Lightsource (SSRL) beamline 12-2 equipped with a Dectris Pilatus 6M detector. Data were indexed, integrated, and scaled using iMosflm, XDS, and Scala.[5–7] Phase information were obtained using the available 1.00 Å resolution structure (PDB: 3U7Q) as a molecular replacement model. Structural refinement, including anisotropic B-factors, and rebuilding were accomplished by using REFMAC5 and COOT, respectively.[8,9] Neutral atomic scattering factors were used in the refinement. As in our previously reported **Av1-CO** structure (PDB: 4TKV), we modeled sulfides in the putative sulfur binding sites 22 Å from the cofactors, although there is no evidence these sulfurs are derived from the belt sulfurs (S2B). Protein structures were displayed in PYMOL, while Mercury was used to illustrate anisotropic B-factors.

CW EPR Spectroscopy. X-band EPR spectra were obtained on a Bruker EMX spectrometer equipped with an ER 4116DM Dual Mode resonator at 10 K using an Oxford Instruments ESR900 helium flow cryostat. Bruker Win-EPR software (ver. 3.0) was used for data acquisition. Spectra were simulated using the EasySpin[10] simulation toolbox (release 5.2.27) with Matlab 2019b.

EPR Av1-CO solution sample preparation. Samples were prepared as 100 μM solutions as frozen glasses in a 50:50 mixture of buffer:ethylene glycol. The buffer solution contained 6.25 mM dithionite ($Na_2S_2O_4$).

EPR crystal slurry sample preparation. Av1-CO crystals were prepared as described above with minor alterations to the crystallization conditions. Crystals were grown in saturated CO solutions of 13-17% PEG 4000 (v/v), 0.5 M NaCl, and 0.2 M imidazole/malate pH 8.0. The crystals were then pooled, washed by centrifugation, resuspended in fresh mother liquor to a final volume of 200 μL, and vortexed to a homogenous slurry. CO was bubbled into the crystal slurry at each step of this process. The slurry was transferred to a homemade gas pressurization device and pressurized at 40 psi of CO for 1 min, then subsequently transferred to an EPR tube, capped, and submerged in liquid nitrogen for storage until EPR data acquisition, resulting in the spectrum shown in **Figure S5**.

Discussion of Previously Reported Spectroscopic Studies with CO: Lo-CO, a CO-bound FeMo-cofactor generated under low CO pressures and hi-CO, a $(CO)_2$-bound FeMo-cofactor, formed under relatively higher CO pressures, exhibit S = 1/2 signals in their EPR spectra, with g = [2.1, 1.98, 1.92] and g = [2.17, 2.06, 2.06] respectively.[11] Signals consistent with these g-values are observed in all of our spectra (**Table S3**). Furthermore, two additional signals, an S = 3/2 signal attributed to the FeMo-cofactor in the as-isolated state of the MoFe protein with g = [4.32, 3.65, 2.01],[12] and an S = 1/2 signal corresponding to the $[4Fe4S]^{1+}$ state of the Fe protein with g = [2.05, 1.94, 1.88][13] were also observed in the **Av1-CO** solution spectra. While the Fe protein can exist in the S = 3/2 and S = 1/2 states, the population of the spin state depends on the solvent conditions. In 50% ethylene glycol, used as a cryoprotectant, most of the Fe protein cluster is in the S = 1/2 state.[14] The CW-EPR spectra were successfully reproduced by including these species in multicomponent simulations with appropriate weighting of all four species in **Figure S3**, or three species in **Figures S4** and **S5**. The generation of either hi-CO and/or lo-CO species (in solution) was dependent on whether CO gas (1 mL) was flushed through the solution before EPR sample

preparation. With respect to **Figure S3**, additional CO was present during EPR sample preparation, while no additional CO was added to the sample corresponding to the EPR spectrum shown in **Figure S4**.

Table S1. Data collection and refinement statistics for **Av1(CO)$_2$** (PDB entry 7JRF). Values in parentheses represent the highest resolution shell.

**Av1(CO)$_2$**	**High Resolution**
**Data Collection Statistics**	
**Wavelength (Å)**	0.99987
**Resolution range (Å)**	39.63 – 1.33 (1.40-1.33)
**Unique Reflections**	447,443 (65,111)
**Completeness (%)**	98.4 (98.1)
**Multiplicity**	5.1 (5.0)
**Space Group**	$P2_1$
**a, b, c (Å)**	77.05 129.97 107.22
**α, β, γ (˚)**	90.00 109.11 90.00
**R_{merge}**	0.085 (0.883)
**$R_{p.i.m.}$**	0.041 (0.434)
**$CC_{1/2}$**	0.998 (0.747)
**I/σ(I)**	10.4 (1.8)
**Data Processing Statistics**	
**R_{work} (%)**	11.60
**R_{free} (%)**	15.00
**r.m.s.d. bond lengths (Å)**	0.016
**r.m.s.d. bond angles (°)**	1.939
**Ramachandran: allowed (outliers) (%)**	99.44 (0.56)

Table S2. B-factors and occupancies of the CO molecules for **Av1(CO)$_2$** (PDB entry 7JRF).

Cofactor ID	CO ID	Binding Mode	Atom	B-factor	Occupancy
ICE A	CMO 3	Bridging	C	6.36	1.0
ICE A	CMO 3	Bridging	O	12.18	1.0
ICE A	CMO 4	Terminal	C	17.80	0.50
ICE A	CMO 4	Terminal	O	20.93	0.50
ICE C	CMO 1	Bridging	C	5.80	1.0
ICE C	CMO 1	Bridging	O	13.15	1.0
ICE C	CMO 2	Terminal	C	17.73	0.50
ICE C	CMO 2	Terminal	O	24.20	0.50

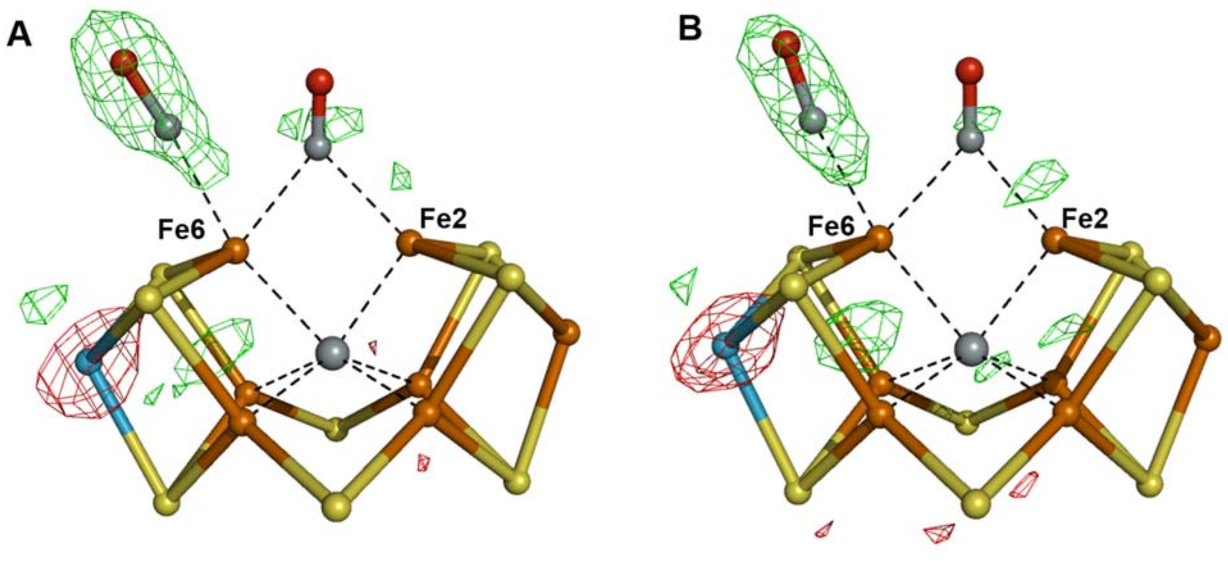

Figure S1. Polder omit maps[15] of terminal CO ligands bound to the **(A)** cofactor of chain A and **(B)** cofactor of chain C. The positive and negative $mF_{\text{obs}} - Df_{\text{model}}$ OMIT difference density is contoured at $\pm$ 3σ and displayed in green and red respectively.

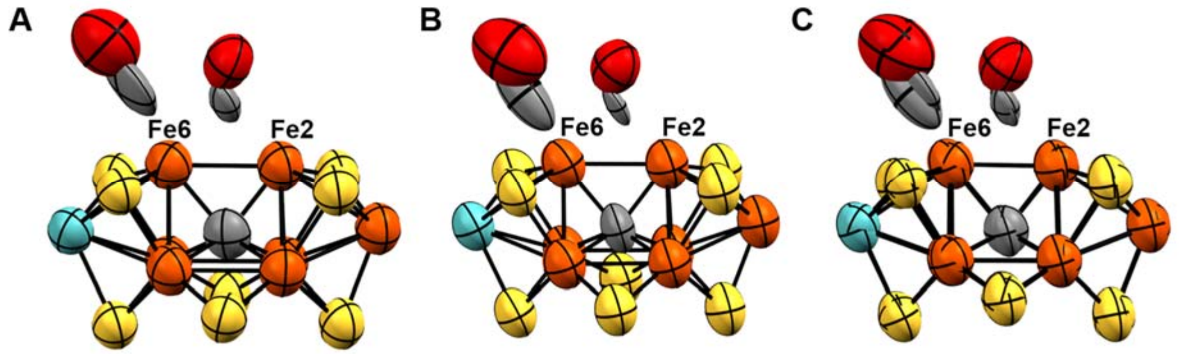

Figure S2. Thermal ellipsoid representation at 50% probability of individually refined anisotropic B-factors for atoms in the FeMo-cofactor and CO. **(A)** FeMo-cofactor located in chain A, with associated CO ligands. These CO ligands are designated in the PDB coordinate file as CMO residues 3 and 4. and **(B)** FeMo-cofactor located in chain C, with CMO residues 1 and 2. **(C)** Superposition of the FeMo-cofactors and associated CO ligands in chains A and C. This figure was calculated with Mercury 2020.2.0.[16]

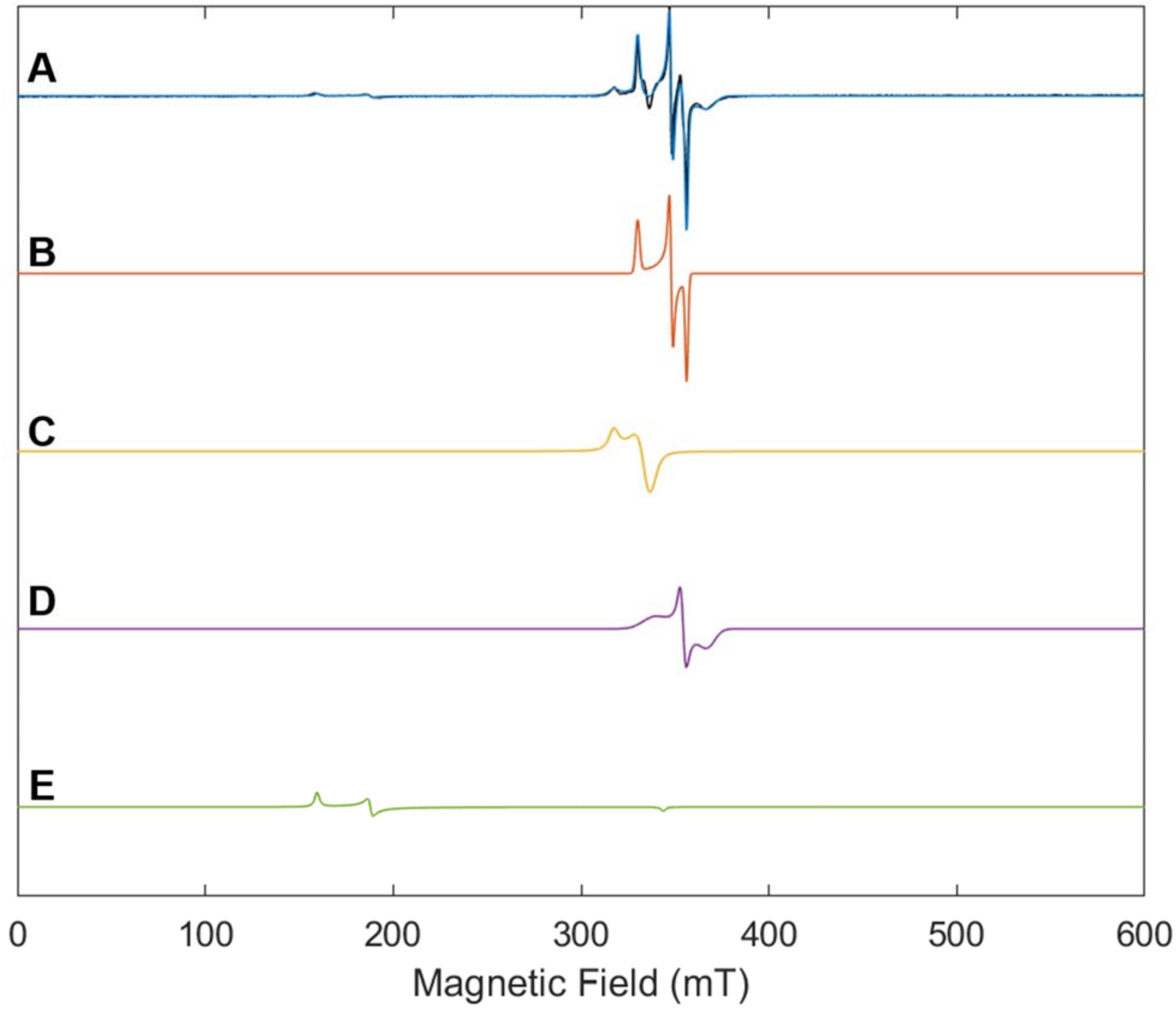

Figure S3. Solution spectrum 1 with **(A)** full simulation (blue) overlaid with experimental data (black), **(B)** lo-CO simulation, **(C)** hi-CO simulation, **(D)** Fe protein simulation, and **(E)** MoFe protein simulation. Frozen solution spectra were collected at 9.64 GHz with a microwave power of 0.5 mW, a modulation amplitude of 9.90 G, a modulation frequency of 100 KHz and conversion time of 82 ms.

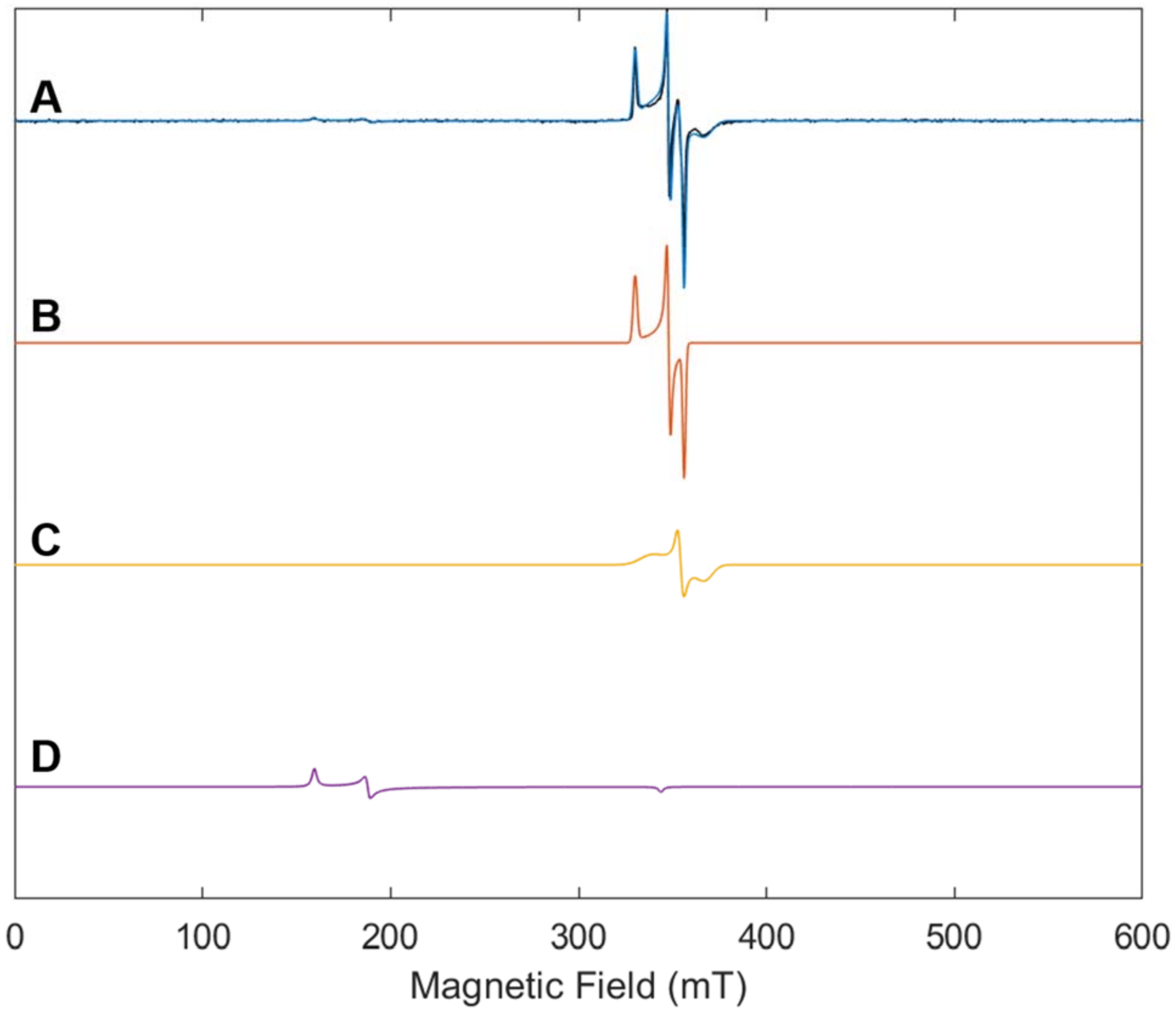

Figure S4. Solution spectrum 2 with **(A)** full simulation (blue) overlaid with experimental data (black), **(B)** lo-CO simulation, **(C)** Fe protein simulation, and **(D)** MoFe protein simulation. Frozen solution spectra were collected at 9.64 GHz with a microwave power of 0.5 mW, a modulation amplitude of 9.90 G, a modulation frequency of 100 KHz and a conversion time of 82 ms.

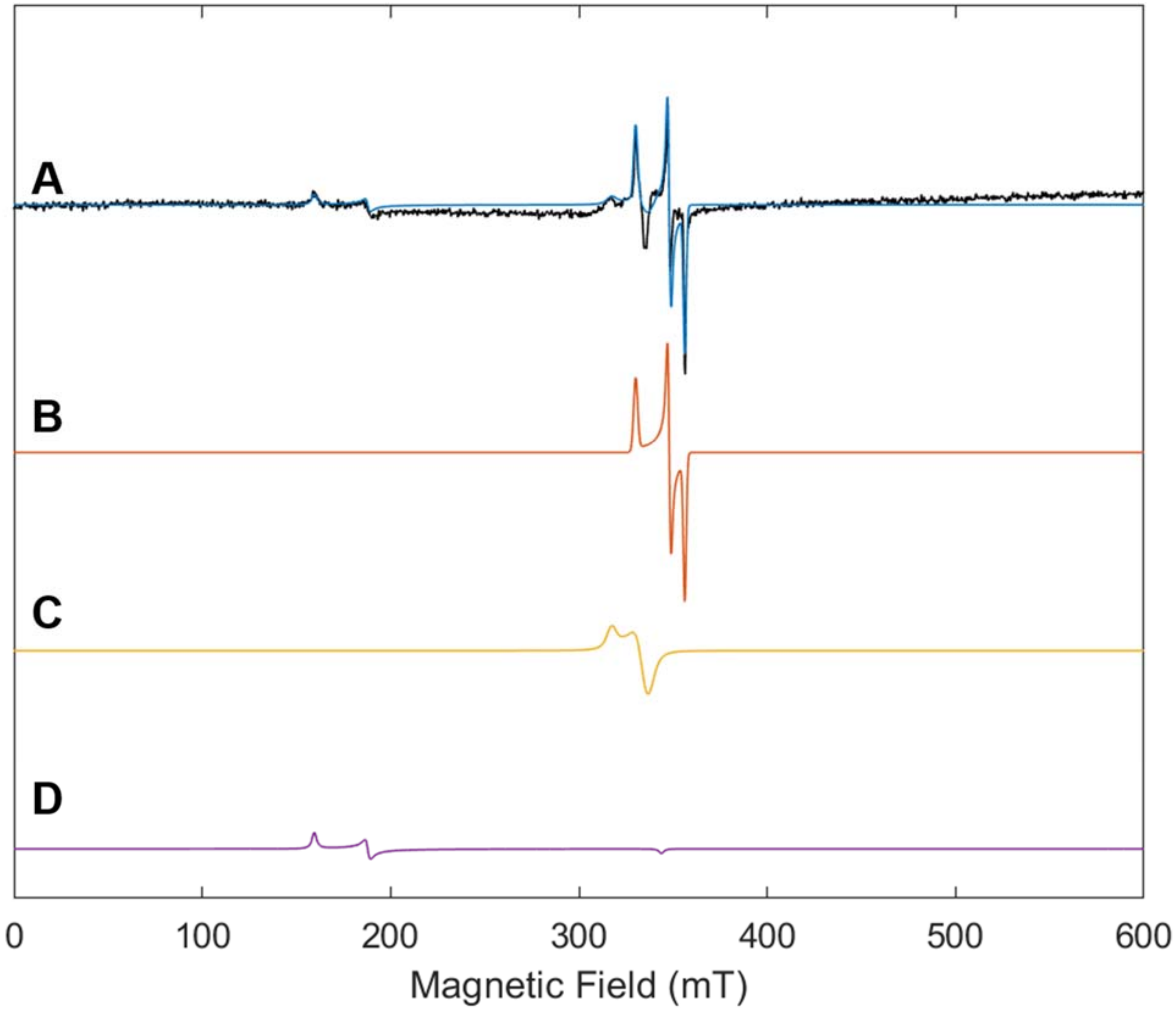

Figure S5. Crystal slurry spectrum with **(A)** full simulation (blue) overlaid with experimental data (black), **(B)** lo-CO simulation, **(C)** hi-CO simulation, and **(D)** MoFe protein simulation. The crystal slurry spectrum was collected at 9.64 GHz with a microwave power of 2 mW, a modulation amplitude of 9.90 G, a modulation frequency of 100 KHz, and a conversion time of 82 ms.

Table S3. EPR Simulation Parameters.

	**Av1**[17]	**Av2**	**lo-CO**	**hi-CO**
S	3/2	1/2	1/2	1/2
g	2.00<br>2.00<br>2.02	2.039<br>1.944<br>1.874	2.086<br>1.979<br>1.933	2.169<br>2.056<br>2.056
**linewidth (Gaussian, Lorentzian) (mT)**	1, 2.5	1	1.8	2, 5
E/D	0.0545	n/a	n/a	n/a
**HStrain (MHz)**	na	400, 70, 250	50, 0.1, 1	50 ,160, 20
**Weight Figure S3A**	0.083	0.333	0.438	0.146
**Weight Figure S4A**	0.098	0.390	0.512	n/a
**Weight Figure S5A**	0.327	n/a	0.531	0.143

Previously reported Av1 simulation parameters were used.[17]

References

1. Spatzal, T. *et al.* Evidence for interstitial carbon in nitrogenase FeMo cofactor. *Science* **334**, 940 (2011).
2. Spatzal, T., Perez, K. A., Einsle, O., Howard, J. B. & Rees, D. C. Ligand binding to the FeMo-cofactor: structures of CO-bound and reactivated nitrogenase. *Science* **345**, 1620–1623 (2014).
3. Spatzal, T., Perez, K. A., Howard, J. B. & Rees, D. C. Catalysis-dependent selenium incorporation and migration in the nitrogenase active site iron-molybdenum cofactor. e*Life* **4**, 11620–11630 (2015).
4. Stowell, M. H. B. *et al.* A simple device for studying macromolecular crystals under moderate gas pressures (0.1–10 MPa). *J. Appl. Cryst.* **29**, 608–613 (1996).
5. Kabsch, W. XDS. *Acta Crystallogr. D Biol. Crystallogr.* **66**, 125–132 (2010).
6. Leslie, A. G. W. The integration of macromolecular diffraction data. *Acta Crystallogr. D Biol. Crystallogr.* **62**, 48–57 (2006).
7. Evans, P. Scaling and assessment of data quality. *Acta Crystallogr. D Biol. Crystallogr.* **62**, 72–82 (2006).
8. Emsley, P., Lohkamp, B., Scott, W. G. & Cowtan, K. Features and development of Coot. *Acta Crystallogr. D Biol. Crystallogr.* **66**, 486–501 (2010).
9. Murshudov, G. N., Vagin, A. A. & Dodson, E. J. Refinement of macromolecular structures by the maximum-likelihood method. *Acta Crystallogr. D Biol. Crystallogr.* **53**, 240–255 (1997).
10. Stoll, S. & Schweiger, A. EasySpin, a comprehensive software package for spectral simulation and analysis in EPR. *J. Magn. Reson.* **178**, 42–55 (2006).
11. Christie, P. D. *et al.* Identification of the CO-binding cluster in nitrogenase MoFe protein by ENDOR of ^{57}Fe isotopomers. *J. Am. Chem. Soc.* **118**, 8707–8709 (1996).

12. Davis, L. C., Shah, V. K., Brill, W. J. & Orme-Johnson, W. H. Nitrogenase II. Changes in the EPR signal of component I (iron-molybdenum protein) of Azotobacter vinelandii nitrogenase during repression and derepression. *Biochim. Biophys. Acta* **256**, 512–523 (1972).

13. Lindahl, P. A., Gorelick, N. J., Münck, E. & Orme-Johnson, W. H. EPR and Mössbauer studies of nucleotide-bound nitrogenase iron protein from Azotobacter vinelandii. *J. Biol. Chem.* **262**, 14945–14953 (1987).

14. Lindahl, P. A., Day, E. P., Kent, T. A., Orme-Johnson, W. H. & Münck, E. Mössbauer, EPR, and magnetization studies of the Azotobacter vinelandii Fe protein. Evidence for a [4Fe-4S]1+ cluster with spin S = 3/2. *J. Biol. Chem.* **260**, 11160–11173 (1985).

15. Liebschner, D. *et al.* Polder maps: improving OMIT maps by excluding bulk solvent. *Acta Cryst. D* **73**, 148–157 (2017).

16. Macrae, C. F. *et al.* Mercury 4.0: from visualization to analysis, design and prediction. *J. Appl. Cryst.* **53**, 226–235 (2020).

17. Morrison, C. N., Spatzal, T. & Rees, D. C. Reversible protonated resting state of the nitrogenase active site. *J. Am. Chem. Soc.* **139**, 10856–10862 (2017).
